# Mucosal and systemic immune responses induced by intranasal immunization of recombinant *Bacillus subtilis* expressing the P97R1, P46 antigens of *Mycoplasma hyopneumoniae*

**DOI:** 10.1042/BSR20191126

**Published:** 2019-10-15

**Authors:** Yongheng Wang, Jialu Wang, Mengyun Zhou, Peng Liu, En Zhang, Yuchen Li, Jian Lin, Zhixin Feng, Qian Yang

**Affiliations:** 1 College of Veterinary Medicine, Nanjing Agricultural University, Weigang 1, Nanjing 210095, Jiangsu, P.R. China; 2 Institute of Veterinary Medicine, Jiangsu Academy of Agricultural Sciences, Key Laboratory of Veterinary Biological Engineering and Technology, Ministry of Agriculture, National Center for Engineering Research of Veterinary Bio-Products, Nanjing 210014, P.R. China

**Keywords:** Bacillus subtilis, intranasal immune, Mycoplasma hyopneumoniae, P46, P97R1

## Abstract

*Mycoplasma hyopneumoniae* (*M. hyopneumoniae*) is the pathogen of swine enzootic pneumonia, a chronic respiratory disease affecting pigs of all ages. The ciliated epithelial cells of the respiratory tract are the main target invaded and colonized by *M. hyopneumoniae.* Therefore, the ideal vaccine would be mucosally administered and able to stimulate suitable mucosal immunity and prevent the adherence of pathogens to mucosal cell surfaces*.* Currently, *Bacillus subtilis* as a recombinant vaccine carrier has been used for antigen delivery and proved to be effectively enhancing the innate immunity of nasal mucosa. Here, our study attempts to construct recombinant *Bacillus subtilis* (B.S-P97R1, B.S-P46), which can express the P97R1 or P46 antigen of *M. hyopneumoniae*, and to evaluate the immune responses in BALB/c mice. Initially, we respectively successfully constructed recombinant B.S-P97R1, B.S-P46 and validated the expression of antigen proteins by Western analysis. Then, recombinant B.S-P97R1 or B.S-P46 were respectively intranasally (i.n.) immunized in mice. Both strong P97R1-specific and P46-specific immunoglobulin G (IgG), secretory immunoglobulin A (SIgA) antibodies were induced in sera, bronchoalveolar lavage fluids (BALs) by ELISA analysis. Moreover, the levels of specific IL-4, IFN-γ in the immunized mice were elevated, and the proliferation of lymphocytes was also enhanced. In general, intranasal inoculation of recombinant B.S-P97R1 or B.S-P46 resulted in strong mucosal immunity, cell-mediated and humoral immunity, which was a mixed Th1/Th2-type response. In addition, our results provided a potential novel strategy that may be applied to the development of vaccines against *M. hyopneumoniae*.

## Introduction

Swine enzootic pneumonia is a chronic respiratory disease caused by *Mycoplasma hyopneumoniae* (*M. hyopneumoniae*) in pigs of all ages [1], which has resulted in enormous economic losses to the swine industry all around the world. Additionally, *M. hyopneumoniae* colonizes and damages the ciliated epithelial cells of the respiratory tract, facilitating other pathogens’ invasion, such as *Pasteurella multocida, Actinobacillus pleuropneumoniae* and virus [2–4]. Currently, the vaccination against *M. hyopneumoniae* is available for inoculation by the intramuscular route, but the effect of this approach has been proved to be not satisfactory [5]. As the target tissue of *M. hyopneumoniae* infections are mucosal sites of the respiratory tract, the most effective strategy to prevent diseases may be through the nasal route [6]. Our previous research showed that attenuated *M. hyopneumoniae* together with bacterial DNA enhanced the local and systemic immune response after intranasal vaccination [7]. However, the reversion of virulence may occur in the live attenuated *M. hyopneumoniae*, and the culture of *M. hyopneumoniae* needs a rich culture medium and a larger time period, increasing the final cost of the vaccine [8].

To develop the new generation of *M. hyopneumoniae* vaccine, several research strategies of an effective vaccine against *M. hyopneumoniae* focus on subunit vaccines [9], DNA vaccines [10] and the utilization of bacterial vectors expressing *M. hyopneumoniae* antigen proteins [11]. Some antigens of *M. hyopneumoniae* have been characterized with immunogenic potential, for instance, the P97 adhesin and its C-terminal region (P97R1), and the 46-kDa membrane surface protein (P46). P97 protein is an important adhesin responsible for the adherence of *M. hyopneumoniae* to respiratory ciliated epithelial cells in swine [12], and has been experimentally tested as vector vaccine’s candidates. Shimoji et al. [13] showed that intranasal immunization with an attenuated strain of *Erysipelothrix rhusiopathiae* YS-19 expressing the C-terminal portion of the P97 protein could not induce antigen-specific immune responses, but can significantly reduce lung lesions caused by *M. hyopneumoniae*. On the other hand, Okamba et al. [12,14] confirmed that intranasal immunized recombinant adenoviruses (*r*Ads) expressing P97(R1) could result in high levels of specific antibodies and significantly reduce the extent of the inflammatory response, compared with intramuscular injection. This finding indicates that the P97 antigen can be protective if administered in a manner that increases its immunogenicity. Additionally, some studies indicate that P46 protein is possibly exposed on the pathogen surface and recognized by the serum of convalescent pig [15]. Therefore, the P46 protein has been tested as DNA vaccines which could induce specific immune responses in mice [10]. However, vector vaccines with the P46 antigen protein have not been investigated.

The ideal vaccine would be mucosally administered and able to stimulate a suitable mucosal immunity, which can prevent the adherence of pathogens to mucosal cell surfaces [16]. However, the previous research mainly focuses on the use of viruses or attenuated bacteria, which leads to increased production costs and the risk of virulence reversion. For the development of a new vaccine for *M. hyopneumoniae*, safety and effectiveness are important considerations. As an internationally recognized safe probiotic, *Bacillus subtilis* (*B. subtilis*) is extensively used as a carrier for expressing the heterologous antigen and induce mucosal immune responses [17,18]. We demonstrated that oral administration of recombinant *B. subtilis* which expresses the S protein of Porcine epidemic diarrhea virus could prevent piglets against Porcine epidemic diarrhea virus infections [19]. Additionally, as a facultative anaerobe, *B. subtilis* is widely distributed in the nasal cavity in pigs [20]. Yang et al. [21] found that the intranasal administration of *B. subtilis* in pigs could enhance the immunity of nasal mucosa to resist respiratory diseases.

The purpose of the present study was to construct recombinant *B. subtilis* which respectively expresses P97R1 or P46 antigen of *M. hyopneumoniae*, and its potential immune responses were evaluated in intranasally (i.n.) immunized mice. We hope to provide a potential novel strategy that may be applied to the development of vaccines against *M. hyopneumoniae*.

## Materials and methods

### Bacterial strains, plasmids

*M. hyopneumoniae* strain 168 was provided by Z.X. Feng (Jiangsu Academy of Agriculture Sciences, Jiangsu, China). *Bacillus subtilis* strain WB800 was obtained from X.W. Gao (Nanjing Agriculture University, Jiangsu, China). pP43NMK plasmid and pLJM1-EGFP (Enhanced Green Fluorescent Protein) plasmid were provided by J. Lin (Nanjing Agriculture University, Jiangsu, China).

### PCR amplification of the EGFP gene, P97R1 gene, P46 gene and site-directed mutation of P46 gene

In our research, the vector pP43NMK was first used, in addition to P97R1, P46 proteins. EGFP was used to determine the function and usability of the pP43NMK.

Genomic DNA of *M. hyopneumoniae* was extracted by Bacterial DNA Kit (Omega) and the plasmid of pLJM1-EGFP was used as a template for the amplification of a 1260-bp fragment (P46 gene), a 250-bp fragment (P97R1 gene) and a 770-bp fragment (EGFP gene). The primers used for amplification were P97R1(F), P97R1(R); P46(F), P46(R); EGFP(F), EGFP(R) (Table 1). They were designed from the previously published sequence of the P97R1 adhesin gene or P46 membrane surface protein gene (GenBank no. U50901) or the instruction of pLJM1-EGFP plasmid.

**Table 1:** The primers information

Primer name	Sequence 5′–3′	Product length
P97R 1 (F)	CATTACCTCAGCCGCCAGCAG	250 bp
P97R 1 (R)	AAGCCATTGGGAAATAGTCT	
P46 (F)	AAATGAAAAAAATGCTTAG	1260 bp
P46 (R)	TTAGGCATCAGGATTATCAACAT	
EGFP (F)	ATGGTGAGCAAGGGCGAGG	770 bp
EGFP (R)	TTACTTGTACAGCTCGTCC	
P46 70 (F)	TCCTCGATGGATTAGTGCC	
P46 70 (R)	GGCACTAATCCATCGAGGA	
P46 101 (F)	AATAACTGGCTCACTCAGC	
P46 101 (R)	GCTGAGTGAGCCAGTTATT	
P46 254 (F)	CCCAGGATGGAATTATGGA	
P46 254 (R)	TCCATAATTCCATCCTGGG	
P97R 1 (F1)	TGTAACACATGCCTCAGCTGCAGCATTACCTCAGCCGCCAGC	290 bp
P97R 1 (R1)	CCATGATTACGCCAAGCTTAAGCCATTGGGAAATAGTCTTCTTTTGG	
P46 (F1)	TGTAACACATGCCTCAGCTGCAGAAATGAAAAAAATGCTTAGAAAAAAATTTTTGTATTCATCAGC	1300 bp
P46 (R1)	CCATGATTACGCCAAGCTTTTAGGCATCAGGATTATCAACATTAGCTTTTGTAACA	
EGFP (F1)	TAACACATGCCTCAGCTGCAGGAATGGTGAGCAAGGGCGAG	810 bp
EGFP (R1)	ATGATTAVGCCAAGCTTTTACTTGTACAGCTCGTCCATGCC	

Underlined are the recognition sites for the restriction enzymes indicated in the table.

**Figure 1:**
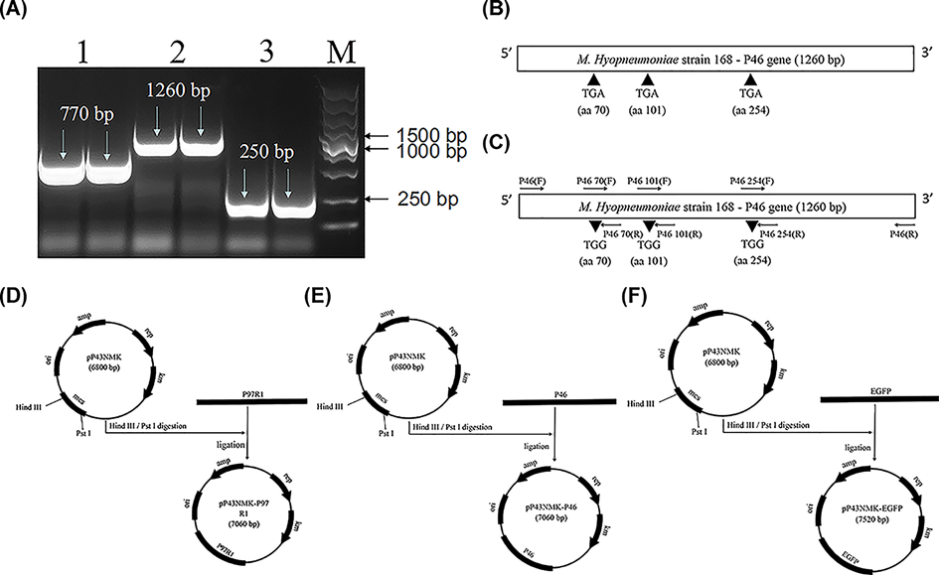
Construction of recombinant *Bacillus subtilis* expressing P97R1, P46 and EGFP proteins (**A**) Identification of the EGFP, P97R1, P46 with PCR. Lanes 1, EGFP gene (770-bp), lane 2, P46 gene (1260-bp); lane 3, P97R1 gene (250-bp). (**B**) Schematic representation of the P46 gene of *M. hyopneumoniae* strain 168 and the positions of TGA codons. (**C**) Schematic representation of site-directed mutagenesis of TGA codons to TGG codons in the P46 gene. The arrows indicate the orientations of the overlapping primers used. P97R1 (**D**) P46 (**E**) EGFP (**F**) fragments were amplified from *M. hyopneumoniae* genome. Three fragments were respectively inserted into the vector pP43NMK to generate the expression vector, pP43NMK-P97R1, pP43NMK-P46, pP43NMK-EGFP.

To express the full-length P46 protein in heterologous cells, TGA codons (tryptophan) in the P46 gene of *M. hyopneumoniae* were replaced with the universal TGG (tryptophan) codons by site-directed mutagenesis using the overlapping extension-PCR method (Figure 1B,C). Amplification reactions were carried out with Phanta® Super-Fidelity DNA Polymerase (Vazyme Biotech Co., Ltd) and primers were listed in Table 1. After amplification, all fragments were sequenced to confirm the correctness of genes.

### Construction of recombinant *B. subtilis* strains

The *B. subtilis* expression vector pP43NMK was chosen to respectively express the P97R1, P46 antigen of *M. hyopneumoniae* and EGFP protein. Briefly, to obtain fragments carrying the vector homologous gene sequence, the 810-bp DNA fragment (EGFP), the 290-bp DNA fragment (P97R1) and the 1300-bp DNA fragment (P46) were obtained by PCR (PCR Thermal Cycler Dice) with the primer pairs EGFP(F1), EGFP(R1); P97R1(F1), P97R1(R1); P46(F1), P46(R1) (Table 1) from three DNA fragments of EGFP, P97R1, P46*.* Then they were respectively inserted into pP43NMK predigested with *Hind*III and *Pst*I, obtaining recombinant plasmids: pP43NMK-EGFP, pP43NMK-P97R1, pP43NMK-P46. Finally, the recombinant plasmids were transformed into *B. subtilis* WB800 by electroporation as previously described [22], the recombinant *B. subtilis* strains were named B.S-EGFP, B.S-P97R1, B.S-P46.

### Analysis expression of EGFP, P97R1, P46 proteins

B.S-EGFP, B.S-P97R1 and B.S-P46 were respectively added into Luria–Bertani (LB) medium for culturing for 16 h, and then washed three times with sterile phosphate-buffered salineant *B. subtilis* strains were observed under Zeiss LSM710 confocal microscope (Zeiss, Oberkochen, Germany) and images were analyzed using ZEN 2012 (Carl Zeiss). The total lysate of *B. subtilis* strain WB800, B.S-EGFP, B.S-P97R1, B.S-P46 were analyzed by SDS/PAGE with the Bio-Rad mini-gel system (12% polyacrylamide gel). The pre-stained protein ladders (YEASEN, China) were used as molecular weight standards. Electrophoretic transfer on to PVDF membranes (Bio-Rad) was done with a Mini-Trans-Blot system (Bio-Rad). The membranes were blocked by incubation with 5% (wt/vol) skim milk in Tris-Buffered Saline with 5% Tween 20 (TBST) for 2 h at room temperature. The mouse anti-P97 antibodies, mouse anti-P46 antibodies (Jiangsu Academy of Agriculture Sciences, Jiangsu, China), and mouse anti-GFP antibodies (diluted to 1:1000 in TBST) were respectively added and incubated overnight at 4°C. The membranes were then washed with TBST three times. The anti-mouse immunoglobulin G (IgG) antibodies conjugated with HRP (Cell Signaling Technology (CST), Inc, America) were diluted 1:5000 in TBST and added to incubate at room temperature for 1 h. Next, the membranes were washed with TBST three times. Then the blots were developed by Super ECL Detection Reagent (YEASEN, China).

### Immunization experiments

BALB/c mice (6 weeks old, specific-pathogen-free [SPF]) were from the Animal Research Center of Yangzhou University (Yangzhou, China). The animal studies were approved by the Ethics Committee for Animal Experimentation of Nanjing Agricultural University. All animal care and use procedures were conducted in strict accordance with the Animal Research Committee guidelines of the College of Veterinary Medicine at Nanjing Agricultural University. The animals’ experiments were performed in Nanjing Agricultural University.

**Figure 2:**
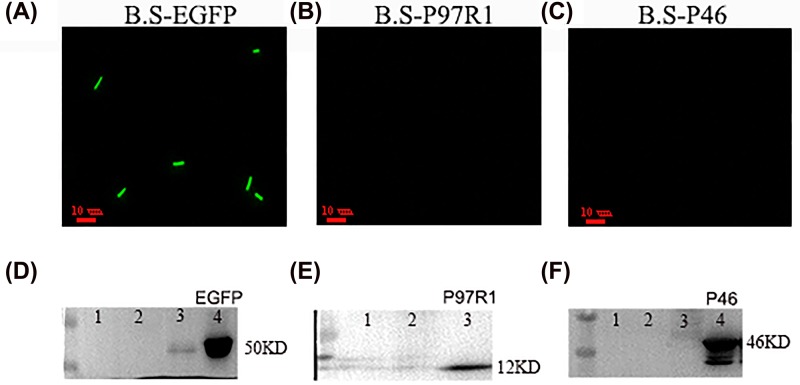
Scheme of intranasal immunization and P97R1-, P46-specific SIgA antibodies response (**A**) Scheme of intranasal immunization, immunization dosage was 20 μl of 5 × 10^8^ CFU of recombinant *B. subtilis* per mouse by intranasal administration. Green represents recombinant *B. subtilis* and primary immunization was performed at 0 day. Booster immunizations were administered at 28 days. The BALs and serum were collected from mice at 0, 7, 14, 28 and 42 days after the first immunization. Additionally, the spleen were collected from mice at 42 days. P97R1-specific SIgA antibodies (**B**) and P46-specific SIgA antibodies (**C**) in the BALs were detected by indirect ELISA. Data are expressed as mean ± *SD*. *P<0.05.

Female BALB/c mice aged 6 weeks were divided into four groups (each group had 20 mice) in the experiments. Group1 was inoculated i.n. with 20 μl of 5 × 10^8^ CFU of *B. subtilis* (B.S) as vector control. Group 2 was treated with 20 μl sterile PBS as negative control (PBS). Group 3 was inoculated i.n. with 20 μl of 5 × 10^8^ CFU of recombinant B.S-P97R1 (B.S-P97R1) and Group 4 was treated with 20 μl of 5 × 10^8^ CFU of recombinant B.S-P46 (B.S-P46) on day zero. Each animal was boosted with the same dose 28 days after the first inoculation. Serum and bronchoalveolar lavage fluids (BALs) samples were collected at 0, 7, 14, 28, 42 days post-inoculation (DPI). The serum and BALs were processed and stored at −20°C until used. The protocol for mice experiment is shown in Figure 2A.

### Immune responses by ELISA analysis

The specific antibodies against P97R1 or P46 were analyzed by ELISA. Briefly, 96-well microtiter plates were respectively coated with 100 μl/well P97R1, P46 proteins (2 μg in 1 ml coating liquid) and incubated at 4°C for 12 h. The wells were then washed three times with PBST (pH 7.4, 150 mM PBS, 0.05% Tween 20) and blocked with 400 μl/well PBS (pH 7.4, 150 mM PBS) containing 5% (wt/vol) skim milk at 37°C for 2 h. The wells were washed three times with PBST, then 100 μl serum (diluted 1:200 in PBST) or 100 μl BALs (diluted 1:50 in PBST) was added to each well, and incubated at 37°C for 1.5 h. Then wells were washed three times with PBST and respectively incubated with 100 μl of 1:2000 diluted horse anti-mouse IgG (or IgG1 or IgG2a) antibodies, rabbit anti-mouse IgA antibodies (CST), which all conjugated with HRP at 37°C for 1 h. The wells were then washed three times and 100 μl of 3,3′-5,5′-tetramethyl benzidine substrate (Solarbio) was added to each well. After 7–15 min of incubation, the reaction was stopped by adding 50 μl of 2 mol/l H_2_SO_4_, and the plates were read at 450 nm with a spectrophotometer plate reader (TECAN).

### Specific lymphocyte proliferation response assay

For assay cell-mediated immune response in mice, splenocytes were prepared as follows. Spleens of mice from each group were removed aseptically at 42 DPI. Splenocytes were isolated using a cell constrainer (Thermo Fisher) and centrifuged at 2000 rpm for 8 min at 4°C. The supernatant was discarded; the cell pellet was resuspended in 0.2 ml of red blood cell lysine to lyse red blood cells for 1 min at 37°C and added with 1 ml sterile PBS to stop the reaction. The solution was centrifuged again and splenocytes were suspended in RPMI 1640 medium (HyClone) supplemented with 10% fetal bovine serum, 100 U penicillin–streptomycin/ml, and 10 μl was removed for cell counting. Then the suspensions were plated in triplicate on to 96-well plates at a concentration of 2 × 10^5^ cells/well and were incubated at 37°C in 5% CO_2_ for 72 h with the corresponding purified P97R1, P46 antigens individually at a final concentration of 10 μg/ml. Concanavalin A (Sigma) (10 μg/ml) was used as a positive control of T-cell stimulation. A 10-μl cell counting kit-8 (CCK8) was added into each well, and cell suspensions were incubated at 37°C in 5% CO_2_ for 3 h, then the plates were read at 450 nm with a spectrophotometer plate reader (TECAN). The lymphocyte proliferation was interpreted as a stimulation index (SI), which is the ratio of 450 nm readings between the stimulated well and the unstimulated one.

### Cytokines measurement

Splenocytes were plated in duplicate on to 24-well plates at a concentration of 3 × 10^6^ cells/well. Cell suspensions were incubated at 37°C in 5% CO_2_ for 72 h with the corresponding purified P97R1 or P46 antigen individually at a final concentration of 10 μg/ml. Then the collected cells were subjected to extraction of total RNA using RNAiso Plus (Takara) and detected mRNA expression of specific IFN-γ, IL-4 cytokines gene. Relative quantification of IFN-γ, IL-4 cDNA to β-actin was conducted on LineGene 9600 Plus (Bioser). RT-PCR primers used in the present study are listed in Table 2. Additionally, the supernatant was collected to detect the concentration of specific cytokines with mouse IFN-γ/ IL-4 ELISA KIT (MULTI SCIENCE).

**Table 2:** The primers’ information for qPCR

Primer name	Sequence (5′–3′)	Product length
IFN-γ-F	TCAAGTGGCATAGATGTGGAAGAA	91 bp
IFN-γ-R	TGGCTCTGCAGGATTTTCATG	
IL-4-F	ACAGGAGAAGGGACGCCAT	94 bp
IL-4-R	GAAGCCCTACAGACGAGCTCA	
β-actin-F	CCCTAAGGCCAACCGTGAA	82 bp
β-actin-R	CAGCCTGGATGGCTACGTACA	

### Statistical analysis

Values for antibodies titers, SI and cytokines production were compared using one-way ANOVA. Results were considered significant for values of *P-*values <0.05.

## Results

### Construction of recombinant *Bacillus subtilis*

To express EGFP, P97R1, P46 proteins in *B. subtilis* WB800, first, EGFP, P97R1, P46 fragments were correctly received by PCR (Figure 1A). Then, three TGA codons were converted into TGG codons by site-directed mutagenesis to ensure full-length expression of P46 in heterologous cells (Figure 1B,C), because the TGA codons encodes tryptophan rather than a translation stop in *M. hyopneumoniae*. Finally, three fragments were respectively inserted into the vector pP43NMK to generate the expression vector pP43NMK-P97R1 (Figure 1D), pP43NMK-P46 (Figure 1E), pP43NMK-EGFP (Figure 1F).

### Electroporation and verification of recombinant *Bacillus subtilis* strains

**Figure 3:**
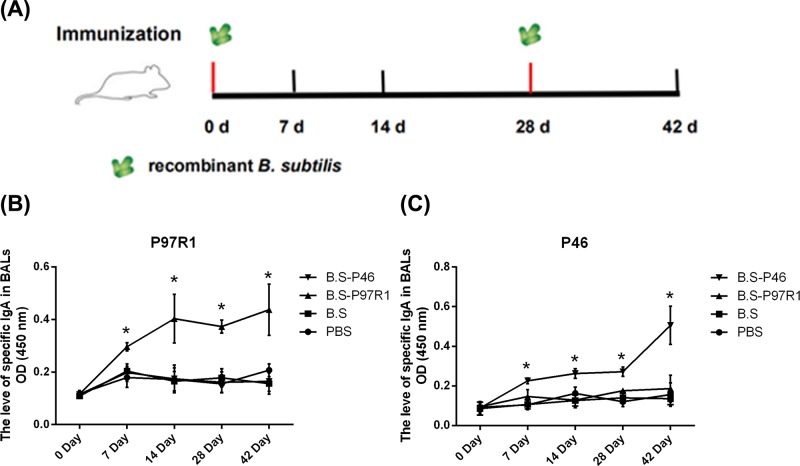
Identify fusion protein EGFP, P97R1, P46 expression Fluorescence microscopy analyzes EGFP, P97R1, P46 protein of B.S-EGFP (**A**), B.S-P97R1 (**B**), B.S-P46 (**C**). The B.S-EGFP (green) was observed. Western blotting using anti-GFP (**D**) lane 1 and 2, *B. subtilis* WB800; lane 3 and 4, B.S-EGFP, protein bands of approximately 50 kDa, anti-P97 (**E**) lane 1, B.S; lane 2, B.S-EGFP; lane 3, B.S-P97R1, protein bands of approximately 12 kDa, anti-P46 (**F**) lane 1 and 2, B.S; lane 3, B.S-EGFP; lane 4, B.S-P46, protein bands of approximately 46 kDa; all bands corresponded to the expected size.

First, to verify whether the recombinant plasmids were successfully imported, we observed the recombinant *B. subtilis* with confocal microscopy. As shown in Figure 3A, the B.S-EGFP was covered by green fluorescence clearly, but B.S-P97R1 (Figure 3B) and B.S-P46 (Figure 3C) could not see green fluorescence which indicated that pP43NMK was useful to construct recombinant *B. subtilis*. To further validate the expression of recombinant *B. subtilis* strains, the target proteins P97R1, P46 and EGFP molecular mass were respectively approximately 50 kDa (Figure 3D), 12 kDa (Figure 3E), 46 kDa (Figure 3F) which could be well detected with the specific antibodies of GFP, P97 and P46 by Western Blot.

### Mucosal immune response elicited by intranasal immunization with recombinant *Bacillus subtilis*

Mice were i.n. immunized with recombinant *Bacillus subtilis* as shown in Figure 2A, and the level of specific secretory immunoglobulin A (SIgA) antibodies in mice was measured by indirect ELISA in the BALs. As shown in Figure 2B, at 7 days, the BALs of the group with B.S-P97R1 contained specific SIgA antibodies against the recombinant P97R1 protein (*P*-value <0.05), then specific SIgA antibodies tended to become steady at 14 days. After booster immunization, the levels of P97R1-specific SIgA antibodies showed a significant increase at 42 days. While the P46 specific SIgA antibodies against the recombinant P46 protein (*P*-value <0.05) were observed in the BALs of the group with B.S-P46 (Figure 2C) and then tended to be steady at 14 days. Additionally, the levels of P46-specific SIgA antibodies showed a significant increase at 42 days after booster immunization. Additionally, there was no cross-reactivity observed.

### Humoral immune response elicited by intranasal immunization with recombinant *Bacillus subtilis*

**Figure 4:**
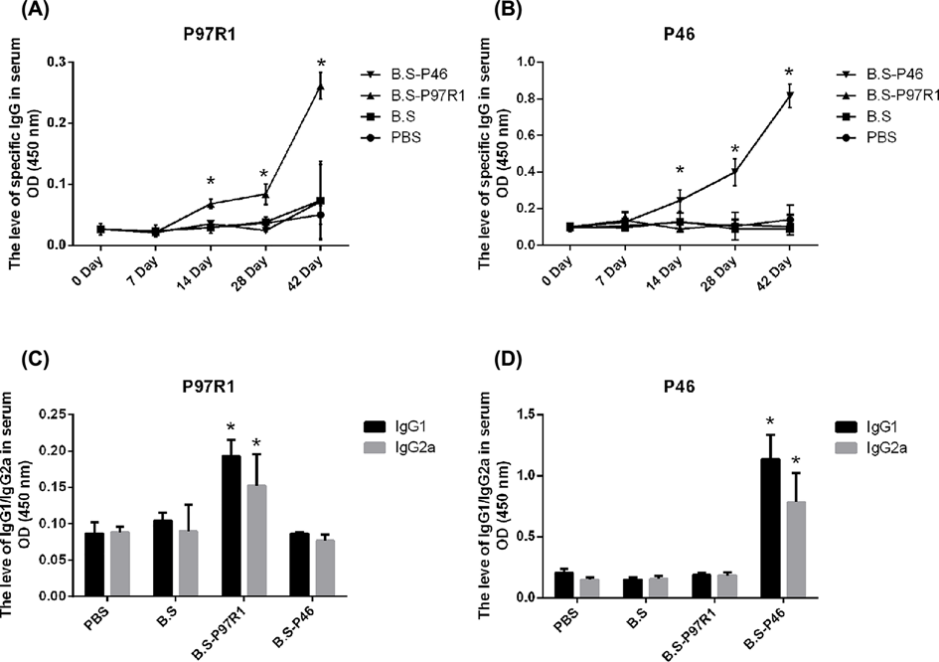
P97R1-, P46-specific IgG antibodies and isotype responses The serum collected from mice at 0, 7, 14, 28 and 42 days after the first immunization were analyzed by P97R1 (**A**), P46 (**B**) specific IgG separately by indirect ELISA. P97R1 (**C**), P46 (**D**) specific IgG subclass in serum were analyzed by indirect ELISA on 42nd day. Data were expressed as mean ± *SD*. *P<0.05.

Additionally, specific P97R1 or P46-IgG antibodies of serum were determined respectively by indirect ELISA. As shown in Figure 4A, the specific IgG antibodies against the recombinant P97R1 protein induced in group with recombinant B.S-P97R1 were significantly higher than other three groups from 14 to 42 days (*P*-value <0.05). While the specific IgG antibodies against the recombinant P46 protein were observed in group with B.S-P46 (Figure 4B) and were also significantly higher than other three groups (*P*-value <0.05).

In mice, IgG1 is indicative of a Th2-type response, whereas IgG2a is predominantly produced during a Th1-type response [23]. In order to determine which Th subset responses were elicited by B.S-P97R1 or B.S-P46, specific IgG isotypes of sera were measured at 42 days (DPI). As shown in Figure 4C,D, the levels of P97R1-specific IgG1 and IgG2a against the recombinant P97R1 protein in group with B.S-P97R1 were significantly higher than other three groups (*P*-value <0.05). In addition, the B.S-P46 had the same comparative results compared with other three groups (*P*-value <0.05).

### Cell-mediated immune response elicited by intranasal immunization with recombinant *B. subtilis*

**Figure 5:**
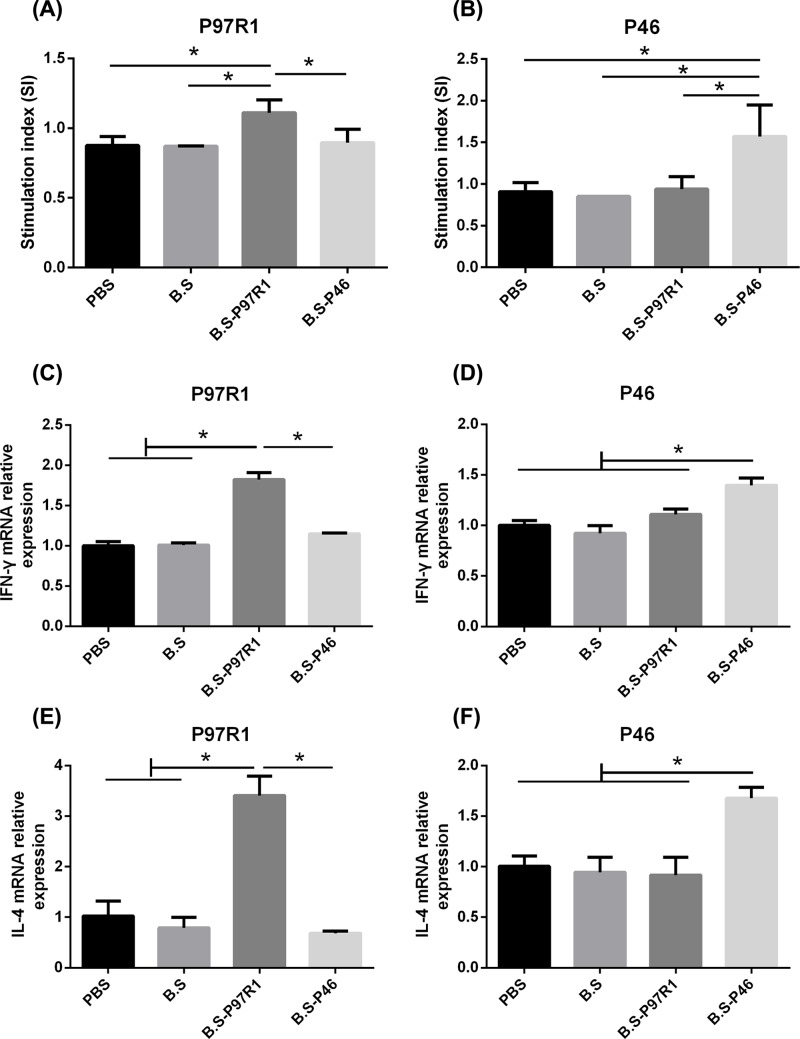
Splenocyte proliferation and the expression of cytokines in responses to P97R1, P46 antigen stimulation Splenocytes were prepared at 42 days and cultured with P97R1 (**A**), P46 (**B**) antigens. Splenocytes proliferation was measured by the CCK-8 method as described in the text and shown as an SI. Data were expressed as mean ± SD. *P<0.05. Cytokine-specific messages from stimulated splenocytes isolated from mice. Splenocytes were prepared and cultured with P97R1 or P46 antigen for 72 h. mRNA expression of cytokines IFN-γ (**C,D**) and IL-4 (**E,F**) were analyzed using RT-PCR. Data were expressed as mean ± *SD*. *P<0.05.

Cell-mediated immune responses play an important role in preventing *M. hyopneumoniae* infections [24,25]. Lymphocytes proliferation responses were detected by CCK8 in the immunized mice at 42 days (DPI). As shown in Figure 5A,B, P97R1-specific and P46-specific proliferation responses were induced in lymphocytes of the mice respectively i.n. immunized with B.S-P97R1 or B.S-P46. The SIs were significantly higher than other groups with B.S (*P*-value <0.05) or PBS (*P*-value <0.05).

### P97R1, P46 modulates the cytokines expression by lymphocytes

**Figure 6:**
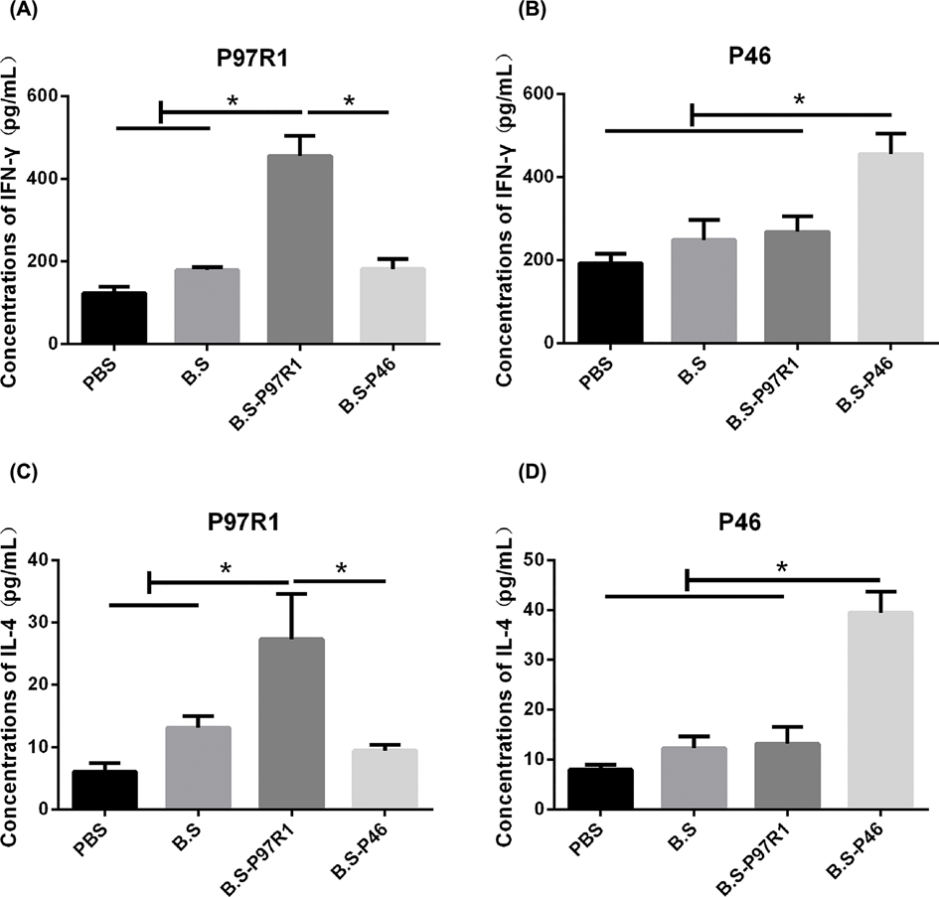
Protein concentrations of cytokines from stimulated splenocytes isolated from mice Splenocytes were prepared and cultured with P97R1, P46 antigen for 72 h. The supernatant was collected for detecting concentrations of specific cytokines IFN-γ (**A,B**) and IL-4 (**C,D**) using mouse IFN-γ, IL-4 ELISA KIT (MULTI SCIENCE). Data were expressed as mean ± *SD*. *P<0.05.

Different cytokines production represents different types of reactions, therefore, splenocytes were re-stimulated with specific P97R1 or P46 antigen respectively *in vitro*. Then the cells were lysed for measurement of cytokines’ mRNAs expression and the supernatant was collected for determination of cytokine concentrations. This is a specific stimulus response. As shown in Figure 5, intranasal administration of B.S-P97R1 or B.S-P46 significantly up-regulated the mRNAs expression of specific IFN-γ (Figure 5C,D), IL-4 (Figure 5E,F) as compared with the PBS, B.S groups (*P*-value <0.05). Moreover, the results of mRNAs expression were in accordance with the results of protein productions as indicated in Figure 6. The concentrations of P97R1-specific and P46-specific IFN-γ (Figure 6A,B), IL-4 (Figure 6C,D) in B.S-P97R1 or B.S-P46 group was significantly higher than other groups (*P*-value <0.05).

## Discussion

As large numbers of immune cells and lymphoid tissues are present in the nasal cavity upper respiratory tract, intranasal vaccination could induce the production of antibodies from lymphoid tissues [26]. The target tissue of *M. hyopneumoniae* infection is mucosal sites of the respiratory tract, therefore, intranasal immunization is an attractive strategy to block entry of *M. hyopneumoniae* and have been applied widely [27]. SIgA is the major effect factors of mucosal immunity in the respiratory tract, and plays a critical role in the defense of mucous membranes against microbial pathogens [28,29]. Some research has shown that SIgA can bind to microbes and prevent them from attaching to or penetrating the epithelial lining, such as *Streptococcus pneumoniae* [30], *Neisseria meningitidis* [31], and *Bordetella pertussis* [32]. In the present study, recombinant B.S-P97R1 i.n. immunized in mice could induce the secretion of specific SIgA on the surface of respiratory mucosal, increasing greatly compared with those in unvaccinated mice. Our results are in agreement with previous studies showing that mucosal immunization with rAdP97c results in both local and systemic immune responses [14]. In addition to the P97R1 protein, P46 protein has also been used as an immunogenic protein for genetic engineering vaccine preparation. Currently, the P46 protein has been used as subunit or DNA vaccines and has been demonstrated that these vaccines could induce higher cell immune response by intramuscular injection, rather than mucosal immunity [10,33]. However, our results indicated that recombinant B.S-P46 could induce higher secretion of specific SIgA on the surface of respiratory mucosa. Additionally, IgG represents systemic immune responses, they also play an important role in maintaining the immune homeostasis [34], participating in opsonization and phagocytosis [35]. We also found that significant P97R1-specific and P46-specific IgG antibodies were induced after intranasal immunization with B.S-P97R1 or B.S-P46, respectively in mice. Our data indicated that B.S-P97R1 or B.S-P46 administrated i.n. respectively may also be beneficial for inducing prolonged and persistent mucosal immunity and humoral responses.

Additionally, it is suggested that an effective immunity against *M. hyopneumoniae* requires cell-mediated immune responses [36]. Both immune responses are driven by the activation of CD4^+^ Th cells. Different types of Th cells determine whether humoral or cell-mediated immunity, and the distribution may be critical for protection against enzootic pneumonia. Th1 cells are involved in the cell-mediated immune response and activate B cells to produce opsonizing antibodies, such as IgG2a, whereas Th2 cells favor humoral immunity and secretion of IgG1 and IgA [37]. Here, we have demonstrated that recombinant B.S-P97R1 or B.S-P46 were respectively capable of inducing both Th1 and Th2 responses to P97R1 or P46, as evidenced by the presence of IgG2a and IgG1 in sera of immunized mice. Interestingly, Conceição et al. [38] reported that mice intranasal immunized with a subunit vaccine containing P97R1 fused to the B subunit of the heat-labile enterotoxin of *Escherichia coli*-induced Th1 immune responses, while given intramuscularly could only induce Th2 responses. Chen et al. [39] immunized orally with the vaccine (*Salmonella typhimurium aroA CS332* carried P97R1) in mice-induced specific IgG antibodies in serum, but not mucosal SIgA, and the vaccine preferentially induced a Th1 response. These research suggested that the different construction, antigens, adjuvant, and route of immunization of vaccines could induce different types of immune responses.

As previously mentioned, respectively intranasally administrated with recombinant B.S-P97R1 or B.S-P46 could stimulate Th cell differentiation and modulate the immune response (IgG1, IgG2a). On one hand, the predominant IgG subclass switch is dependent on T cells, on the other hand, cytokines of T cells also play an important role in it [40]. It is now clear that Th1 cells mainly secret IFN-γ, while Th2 cells secret IL-4 in mice [41]. After intranasal immunization, we observed that recombinant B.S-P97R1 or B.S-P46 could respectively induce significant P97R1-specific or P46-specific IFN-γ, IL-4 production compared with the control groups. Furthermore, IFN-γ secreted in the respiratory tract and lungs can kill pathogens and has been shown to be important for macrophage activation in the respiratory tract to prevent *M. hyopneumoniae* infection [6]. In addition, P97R1 or P46 antigen could respectively significantly stimulate specific lymphocyte proliferation responses of immunized animals *in vitro*. All these results demonstrated that recombinant B.S-P97R1 or B.S-P46 could well induce cell-mediated immune responses.

In conclusion, the present study described the successful constructions of recombinant B.S-P97R1 and B.S-P46, evaluated the immune effect of i.n. immunized mice. The levels of BALs SIgA, serum IgG, the proliferation of splenocytes and the levels of T-cell-specific cytokines significantly increased in mice respectively i.n. immunized with recombinant B.S-P97R1 or B.S-P46. The present work demonstrates that the recombinant B.S-P97R1 or B.S-P46 may respectively induce local mucosal, humoral and cellular immunities and represents a potentially new approach to design vaccine against *M. hyopneumoniae*. However, the effectiveness of recombinant *B. subtilis* for the control of swine enzootic pneumonia requires further studies in piglets.

## Abbreviations

BALs: bronchoalveolar lavage fluid
B.S: *Bacillus subtilis*
CCK8: cell counting kit-8
CFU: colony-forming units
DPI: day post-inoculation
EGFP: enhanced green fluorescent protein
HRP: horseradish peroxidase
IFN: interferon
IgG: immunoglobulin G
IL: interleukin
i.n.: intranasally
PBS: phosphate-buffered saline
SI: stimulation index
SIgA: secretory immunoglobulin A
TBST: tris-buffered saline with 5% tween 20
